# The Impact of Economic Distress on Primary Headache Visits Under the Strain of the COVID-19 Pandemic: A Retrospective Study

**DOI:** 10.3390/jcm15114181

**Published:** 2026-05-28

**Authors:** Merih Can Yilmaz, Ozgur Ozaydin, Keramettin Aydin

**Affiliations:** 1 Department of Neurosurgery, VM Medical Park Hospital, 55200 Samsun, Turkey; keramettinns@gmail.com; 2 Department of Economics, Ondokuz Mayis University, 55270 Samsun, Turkey; ozaydin@omu.edu.tr

**Keywords:** socioeconomic factors, inflation, unemployment, financial toxicity, primary headache

## Abstract

**Background and Objectives**: Macroeconomic instability, particularly income loss, inflation and unemployment, is increasingly recognized as a psychosocial stressor that may influence both symptom burden and healthcare-seeking behavior. This single-center study investigated the association of income, inflation and unemployment with private-sector hospital visits for primary headache disorders and assessed whether economic stressors were associated with different patterns across demographic groups. **Materials and Methods**: We conducted a single-center, retrospective, ecological quarterly time-series analysis of hospital visits for primary headache disorders between 2016 and 2024 in a private tertiary care hospital in Turkey. After exclusions, 18,522 eligible hospital-visit records were included and categorized by sex and age (<18, 18–64, and ≥65 years). National data on real gross domestic product (GDP), consumer price index (CPI), unemployment and a COVID-19 period indicator were used. Counts were modeled with log-linked Poisson or negative binomial generalized linear models selected through overdispersion diagnostics, with seasonal controls and HAC-robust inference. **Results**: In most groups, higher GDP was associated with more primary headache visits, whereas higher inflation was consistently associated with fewer visits. The association with unemployment was heterogeneous: visits decreased significantly among the working-age population but increased among older adults. Contemporaneous models outperformed one-quarter lagged alternatives, suggesting that private-sector healthcare seeking may change within the same quarter as macroeconomic shocks. **Conclusions**: In this private hospital setting, macroeconomic deterioration was associated with reduced primary headache visits, particularly among working-age patients. These findings suggest that financial constraints may suppress private-sector healthcare utilization despite possible increases in stress-related symptoms, and that private hospital data may underestimate headache-related healthcare need during economic crises.

## 1. Introduction

In recent years, the psychosomatic and healthcare utilization consequences of economic hardship have received increasing attention in medicine, psychology and public health [1,2,3]. Macroeconomic factors such as income, unemployment and inflation may affect financial stability and may also influence physical and mental health [4,5,6]. Financial toxicity, a term proposed by Zafar and colleagues to describe the objective financial burden and subjective financial distress related to healthcare costs, has increasingly been used to explain delayed care, avoidance of medical services and reduced treatment adherence [7,8]. In developing countries, where economic turmoil can increase social vulnerability, these stressors may be particularly intense.

Primary headache disorders are common and clinically important conditions that may be influenced by psychosocial stressors [9,10]. Migraine and tension-type headache are major primary headache disorders classified by the International Classification of Headache Disorders, 3rd edition (ICHD-3) [10]. Recent global evidence indicates that active headache disorders affect approximately 52.0% of the population, migraine 14.0%, tension-type headache 26.0%, and headache occurring on 15 or more days per month 4.6% [11]. Migraine also contributes substantially to disability and health-system burden worldwide [12]. In Turkey, migraine was diagnosed in 16.4% of adults in a nationwide home-based study, and tension-type headache was diagnosed in 5.1% [13]. A recent school-based study from Istanbul also showed that headache disorders are common among children and adolescents, supporting the need to consider age-specific patterns in Turkey [14].

Primary headache disorders may be associated with psychological stressors such as depression, anxiety and socioeconomic fluctuations. Previous studies have reported associations between economic crises and sleep disturbances, mood disorders, and somatic complaints in Turkey [15,16,17] and internationally [18,19,20,21,22,23]. However, research specifically focusing on the relationship between macroeconomic indicators such as income, unemployment and inflation, and private-sector visits for primary headache disorders remains limited.

This study examines the association of income, unemployment and inflation with quarterly private-sector hospital visits for primary headache disorders in Turkey’s Black Sea region. The primary research question was whether quarterly macroeconomic indicators were associated with changes in private-sector visits for primary headache disorders. We hypothesized that macroeconomic stressors would be associated with changes in primary headache visits, but that the direction of the association in the private sector might reflect a balance between stress-related symptom burden and financial constraints that limit care seeking. Patients with secondary headache causes, including trauma, tumors, upper respiratory tract infections and cerebrovascular events, were excluded.

## 2. Materials and Methods

This was a single-center, retrospective, ecological, quarterly time-series study using a biopsychosocial framework to examine the direction and magnitude of the association between economic stressors and hospital visits for primary headache disorders. The analysis assessed whether reduced purchasing power and labor-market instability were associated with changes in private-sector healthcare utilization. National-level economic data were used to operationalize the environmental burden, based on the assumption that in highly centralized economies such as Turkey, inflation shocks and labor market volatility may function as systemic environmental stressors rather than only local ones. Because the study period included the COVID-19 pandemic, a COVID-19-period indicator was included to assess whether the outbreak coincided with additional changes in primary headache visits. Ethical approval was obtained from the Institutional Ethics Committee of Ondokuz Mayis University Faculty of Medicine Hospital, application number 2025/422, approval date 5 September 2025. During the preparation of this manuscript, the authors used ChatGPT-5.5 for English language editing.

The retrospective ecological quarterly protocol was selected because the research question concerned quarter-level changes in private-sector utilization in relation to macroeconomic indicators, which are reported at quarterly frequency. The protocol did not involve validation of a clinical scale or questionnaire; instead, it was based on predefined eligibility and exclusion criteria, quarterly aggregation, and prespecified count-model diagnostics. Reporting was reviewed against the STROBE checklist for observational studies, and a point-by-point checklist is provided as Supplementary Table S4.

### 2.1. Structural Framework

To empirically measure the association between environmental load and primary headache visits, the following model was used, in which visit frequency was evaluated as a function of core economic indicators.

$$A=f(Y,SED,PP,d_{COV})$$

where A represents the quarterly frequency of hospital visits due to primary headache disorders. The independent variables were selected as indicators of different dimensions of economic stress: income (Y) represents general economic health and standard of living; socioeconomic deprivation (SED) reflects the anxiety induced by the dual burden of financial hardship and the loss of social status; purchasing power (PP) acts as an immediate stressor affecting the daily cost of living; and the COVID-19-period indicator (dCOV) captures the structural shock of the COVID-19 pandemic and the associated psychological and socioeconomic stress experienced during this period.

### 2.2. Data Collection

The data were derived from the hospital information system of Samsun Medical Park, a large private tertiary healthcare center serving Turkey’s Black Sea region. The study period covered 2016–2024, a period of considerable economic fluctuation across the country. The unit of analysis before quarterly aggregation was the hospital-visit record rather than a confirmed inpatient stay.

The raw dataset included 18,589 hospital-visit records in which headache was the primary complaint and a primary headache disorder was supported by ICD-10 (International Statistical Classification of Diseases and Related Health Problems) coding and physician notes. Eligible records were required to be clinically compatible with the ICHD-3 framework for primary headache disorders, including migraine, tension-type headache, trigeminal autonomic cephalalgias and other primary headache disorders, and not better accounted for by another secondary headache diagnosis. The clinical eligibility logic and exclusion criteria were reviewed by a neurologist before submission. Visits with evidence of secondary headache causes, including trauma, tumors, upper respiratory tract infections and cerebrovascular events, were excluded before constructing the analytic dataset.

Prior to analysis, 67 records from children aged 5 years and younger (0.36% of the total) were excluded in accordance with ICHD-3 considerations regarding the reliability of self-reported primary headache symptoms in preschool children with limited verbal expression.

**Figure 1:**
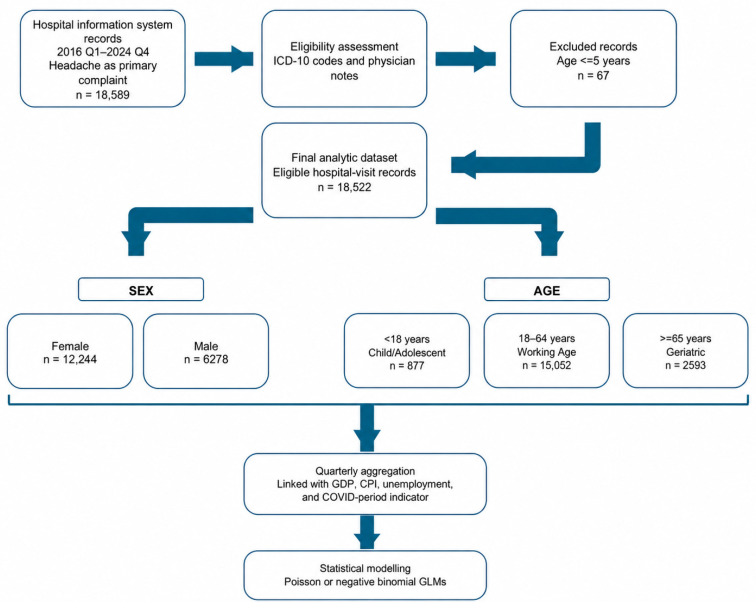
Flowchart of research design and analytic workflow.

The final analytic dataset comprised 18,522 eligible hospital-visit records. In accordance with the periodicity of macroeconomic data, visit counts were converted into quarterly time series using Python (v3.13.9). As there were no missing observations, negative values or zero counts, data completion was not required in the final dataset. Figure 1 presents the overall research workflow, including record identification, exclusion criteria, construction of the final analytic dataset, demographic stratification, and quarterly aggregation for statistical modeling.

#### 2.2.1. Stratification Strategy

To study the different associations of economic stressors across demographic groups, the study population was grouped by age and sex. The 18,522 eligible hospital-visit records were categorized into three age groups: <18 years (Child/Adolescent), 18–64 years (Working Age) and ≥65 years (Geriatric). This stratification was used to capture potential differences in exposure to labor-market shocks, dependency status and care-seeking behavior.

Although the OECD definition of the working-age population is 15–64 years, the working-age cohort was defined as 18–64 years because of the 12-year compulsory education system in Turkey and the limited labor market participation of the 15–17 age group. This definition also aligns more closely with the medical age grouping used in this study.

#### 2.2.2. Macroeconomic Data Sources

Independent variables were obtained from the Turkish Statistical Institute (TurkStat) database covering 2016’s first quarter to 2024’s fourth quarter. GDP was used as the chained volume index (2009 = 100) to adjust for inflation and reflect real economic growth; CPI was used as the general consumer price index based on 2003 = 100 prices, representing the loss of consumer purchasing power; and unemployment was measured using the unemployment rate for persons aged 15 years and older to reflect labor-market conditions.

### 2.3. Modeling Framework

We modeled the outcomes as count processes using generalized linear models (GLM) with a log link, estimated by maximum likelihood [24]. The baseline specification was a Poisson regression, which implies equality of the conditional mean and conditional variance [25]. To accommodate potential extra-Poisson variation, we allowed for a negative binomial alternative (NB2), parameterized with a dispersion parameter that governs the extent to which the variance exceeds the mean [26,27].

#### 2.3.1. Covariate Specification, Transformations, and Timing

All models were estimated with a pre-specified contemporaneous covariate set gross domestic product (GDP), unemployment, and the consumer price index (CPI), together with a binary COVID-19-period indicator (coded 1 from 2020Q2 onward and 0 otherwise). The macroeconomic covariates entered the model in logarithmic form, whereas the dependent variable was kept in its raw count scale to preserve the discrete distributional interpretation underlying Poisson/NB likelihoods [28].

Prior to estimation, the stationarity of the logarithmic macroeconomic series was evaluated using the Kwiatkowski–Phillips–Schmidt–Shin (KPSS) test [29]. This diagnostic step is performed to verify whether the series are trend-stationary or stationary to avoid the risk of misleading regression results.

We applied the simultaneous identification method based on the principle of frugality for macroeconomic indicators. With moderate sample size, introducing lag structures for multiple regressors would materially reduce degrees of freedom, increasing the risk of overparameterization and inflating estimator variance [30]. In addition, because the data are aggregated at the quarterly level, this temporal aggregation supports interpreting the contemporaneous covariate values as capturing the dominant within-quarter adjustment. To empirically validate this argument, we conducted preliminary sensitivity analyses using 1-quarter lags. Consistent with our theoretical framework, these alternative specifications yielded structurally similar results but exhibited reduced goodness-of-fit, as evidenced by higher AIC and BIC values (see Supplementary Table S1); thus, the contemporaneous specification was retained.

#### 2.3.2. Seasonality Control

To control quarterly seasonality, quarter-of-year indicators were included in all models, with Q1 serving as the reference category to avoid perfect collinearity [31].

#### 2.3.3. Handling of Exposure and Offset

Although count data models often employ a population offset to model rates, we modeled raw counts directly. First, reliable population measures were available only at an annual frequency, and mechanically interpolating them (e.g., via linear or cubic splines) would introduce smooth, non-stochastic trends that can distort inference. Second, demographic change is slow-moving relative to macroeconomic shocks; thus, the population effect can be treated as approximately time-invariant and absorbed by the model intercept [25]. Furthermore, throughout the study period, the hospital’s bed capacity, major insurance agreements, and catchment area characteristics remained structurally stable, minimizing the risk of exogenous demand shifts unrelated to macroeconomic conditions.

#### 2.3.4. Screening for Overdispersion and Eligibility for Negative Binomial Estimation

After fitting the Poisson model, overdispersion was evaluated using three complementary diagnostics: the Pearson dispersion statistic (phi), for which values materially above one indicate overdispersion [24]; the Cameron–Trivedi-type dispersion test, an auxiliary-regression-based test designed to detect variance exceeding the Poisson restriction [32]; and the Dean-type test, a standardized diagnostic for extra-Poisson dispersion [33]. NB estimation was pursued only when formal tests were significant (*p* < 0.05) and phi > 1.10.

#### 2.3.5. Poisson Versus NB Selection

When an NB model was eligible, we compared Poisson and NB via a likelihood-ratio (LR) test. Since the null hypothesis (dispersion = 0) places the parameter on the boundary of the parameter space, we used a boundary-adjusted *p*-value [34]. Model choice followed a structured rule: NB was selected when the LR test supported NB (*p* < 0.05); if the LR test was borderline, AIC [35] and BIC [36] were used as information-criterion tie-breaks, otherwise Poisson was retained.

#### 2.3.6. Inference Using HAC-Robust *p*-Values

For the selected model, coefficient-level inference was based on heteroskedasticity- and autocorrelation-consistent (HAC) *p*-values computed using a score-driven Newey–West sandwich estimator with Bartlett weights [37]. The lag truncation was set up to four lags [38].

#### 2.3.7. Model Adequacy Checks via Randomized Quantile Residuals

Model adequacy was assessed using randomized quantile residuals (Dunn–Smyth residuals), which are designed to behave as standard normal variates under correct specification [39]. Diagnostics included a Kolmogorov–Smirnov test for normality [40], Ljung–Box tests for serial correlation [41], and visual inspection of ACF and Q-Q plots.

## 3. Results

### 3.1. Descriptive Statistics and Sample Characteristics

The analysis covered 36 consecutive quarters from 2016 Q1 to 2024 Q4, a timeframe marked by substantial macroeconomic volatility. The final analytic dataset consisted of 18,522 eligible hospital-visit records, with a quarterly visit mean of 514.50 (SD = 112.30). Distribution of the study population by age group and sex is presented in Table 1, and the patient-level descriptive statistics for quarterly clinical visit counts and raw macroeconomic indicators are summarized in Table 2.

### 3.2. Model Selection

Following the multi-step diagnostic procedure detailed in the Materials and Methods section, the appropriate count distribution for each demographic stratum was determined by evaluating overdispersion diagnostics.

As presented in Table 3, significant overdispersion was identified in the Overall, Female, Male, and Working Age cohorts. In these groups, not only did the Pearson dispersion statistic phi exceed the 1.10 threshold, but both Cameron–Trivedi and Dean’s tests also yielded significant results (*p* < 0.05), with subsequent likelihood-ratio tests confirming the superiority of the negative binomial specification (*p* < 0.001).

Conversely, Poisson models were retained for the remaining groups based on the prespecified diagnostic rule. For the <18 years group, although the Pearson phi was 1.74, the CT (*p* = 0.34) and Dean (*p* = 0.16) tests did not provide formal statistical evidence of overdispersion. For the ≥65 years group, the LR test was borderline (*p* = 0.06); therefore, the Poisson model was retained as the more conservative specification with similar information criteria compared with the NB alternative.

To assess the robustness of these decisions, the <18 years and ≥65 years groups were also estimated using a negative binomial framework. These sensitivity analyses produced structurally similar results. The findings of these alternative specifications are given in Supplementary Table S2.

The better fit of the contemporaneous model compared with the one-quarter lagged model [evidenced by lower AIC and BIC values across demographic strata (Table S1)] suggests that private-sector healthcare seeking may change within the same quarter as macroeconomic shocks.

### 3.3. Estimation Results

According to the prespecified diagnostic procedures and model-selection criteria, models were estimated for the overall sample and demographic groups; the results are presented in Table 4.

The estimation results show that GDP was positively and statistically significantly associated with visits in all demographic groups at the 1% level. The coefficient reached its highest value in the <18 years group (6.08, *p* < 0.01), suggesting that income growth may be related to greater use of private health services. In contrast, CPI, representing inflation and loss of purchasing power, yielded negative and significant coefficients in all models, suggesting that inflation may suppress private-sector visits across groups.

The association between unemployment and visits differed across age groups. In the working-age population (18–64 years), higher unemployment was associated with fewer visits (β = −0.61, *p* < 0.01). In contrast, unemployment was positively associated with visits among older adults (≥65 years; β = 0.69, *p* < 0.01). In the male and child/adolescent groups, the unemployment coefficient was not statistically significant.

The dummy variables included in the models showed seasonal patterns and pandemic-period changes that differed across demographic groups. Compared with Q1, Q2 was associated with fewer visits among men, children/adolescents and older adults, as in the overall sample. The Q2 coefficient was not statistically significant among women or the working-age population. The COVID-19-period indicator had a negative coefficient in all estimated models, but this association reached marginal statistical significance (*p* < 0.10) only among women.

### 3.4. Residual Diagnostics

Model adequacy was assessed through randomized quantile residuals (RQR). Kolmogorov–Smirnov test results (*p* > 0.05) across all demographic groups indicated no major departure from the expected standard normal residual distribution. Ljung–Box tests also suggested no residual serial correlation (*p* > 0.05). Detailed diagnostics are presented in Supplementary Table S3.

## 4. Discussion

The present findings should be interpreted primarily as changes in private-sector healthcare utilization rather than as direct evidence of changes in the underlying incidence or burden of primary headache disorders. Recent evidence suggests that inflation has become an important source of psychosocial stress, particularly among socioeconomically vulnerable groups, and that healthcare-related financial toxicity is associated with delayed care, forgone care, and cost-related medication nonadherence [42,43]. This framework is especially relevant to migraine and other primary headache disorders, which have been associated with substantial healthcare resource utilization and cost burden in recent registry- and claims-based studies [44,45]. Therefore, the observed decrease in private-sector headache visits during periods of inflationary and economic stress may reflect cost-sensitive care seeking, deferred care, self-management, or substitution toward lower-cost services rather than a true reduction in headache burden. However, these mechanisms were not directly observed in the present study and should be examined in future research incorporating patient-level socioeconomic data, public-sector utilization records, and medication-use information.

Financial stress has also been linked with healthcare avoidance and poorer psychological outcomes in other populations. For example, studies of financially pressured trainees and employed adults indicate that inflation-related or debt-related stress may coincide with delayed medical care, poorer mental health, anxiety, sleep problems and changes in work-related stress exposure [46,47]. Although these studies were not specific to headache disorders, they support the broader interpretation that macroeconomic strain may alter both symptom burden and decisions to seek care.

Earlier economic-psychology and occupational studies further suggest that inflation is not merely a financial indicator but also a perceived stressor that can shape satisfaction, organizational attitudes and wellbeing [48,49,50,51,52,53,54]. In this context, the negative association between CPI and private-sector headache visits in the present study should not be read as evidence that inflation reduces headache burden. Rather, it is more consistent with the interpretation that the utilization-suppressing effect of reduced purchasing power may outweigh any stress-related increase in symptoms in a private hospital setting.

The unemployment findings should be interpreted against a large literature backdrop linking joblessness with mental and physical health. Prior studies and reviews have associated unemployment with depression, anxiety, suicidal behavior, cognitive strain, glucose–metabolism disorders and immune-function changes [55,56,57,58,59,60,61,62,63]. Our working-age results are consistent with the possibility that unemployment reduces private-sector healthcare use through liquidity preservation, insurance or employment-related access pathways, and postponement of non-emergency care. The positive association observed among older adults may reflect different household, retirement-income or caregiving pathways, but these mechanisms remain inferential because individual income, insurance status and household composition were unavailable.

Finally, the findings remain clinically plausible within psychosomatic models of primary headache. Chronic headache, migraine and tension-type headache have been linked to stress, depression, anxiety, medication overuse and other psychosocial factors [64,65]. The present study extends this literature by suggesting that macroeconomic stressors may affect observed private-sector headache visits through a dual pathway: they may increase stress-related symptom pressure while simultaneously reducing the ability or willingness to use private healthcare.

Taken together, this pattern can be interpreted through the lens of financial toxicity while distinguishing clinical incidence from effective demand for private healthcare. A decline in private-sector visits should not be interpreted as a reduction in the true prevalence of stress-related headaches in the general population. Instead, economic distress may increase symptom burden while simultaneously reducing private-sector utilization because patients defer care, self-medicate or seek lower-cost public-sector alternatives. The better fit of the contemporaneous models compared with the lagged specifications (Table S1) further suggests that these utilization changes may occur rapidly within the same quarter as macroeconomic indicators worsen.

### Policy Implications

The results suggest that during economic recessions, private hospital records may not fully reflect population-level headache burden because financial toxicity can suppress private-sector utilization. Policymakers and clinicians should consider the possibility of deferred care or shifts toward public services during periods of high inflation and unemployment. However, because this study did not include public-sector data, the substitution hypothesis should be regarded as a plausible explanation that requires confirmation in multicenter datasets including both private and public providers.

This study has several limitations. First, data were collected from a single private hospital, which allows focused analysis of private-sector utilization but limits generalizability to public hospitals and other regions. Second, because of the retrospective ecological design and the use of aggregate macroeconomic indicators, the analysis cannot establish patient-level causality. Individual income, insurance status, out-of-pocket payments, headache subtype severity and public-sector visits were not available. Third, while we hypothesize that some patients shifted toward public care or self-management, this pathway was not directly observed. Future multicenter studies integrating private and public datasets with patient-level socioeconomic variables are needed.

## 5. Conclusions

In this single-center retrospective time-series study, macroeconomic deterioration was associated with fewer private-sector visits for primary headache disorders, particularly among working-age patients. Older adults showed a different pattern, with unemployment positively associated with visits. These findings suggest that private hospital records may underestimate headache-related healthcare need during economic crises because patients may defer care, self-manage symptoms, or seek lower-cost alternatives. Further multicenter studies including public-sector data and patient-level socioeconomic variables are needed to confirm these mechanisms.

## Figures and Tables

**Table 1:** Distribution of the study population by age group and sex.

Total Sample	18,522 (100.0%)
Age group	
<18 years, Child/Adolescent	877 (4.7%)
18–64 years, Working Age	15,052 (81.3%)
≥65 years, Geriatric	2593 (14.0%)
Sex	
Male	6278 (33.9%)
Female	12,244 (66.1%)

**Table 2:** Descriptive statistics.

Variable Category	Cohort	Mean	Median	Std. Dev.	Skewness	Kurtosis	Jarque–Bera	Probability
**Visits** **(Counts)**	Overall	514.50	513	89.91	−0.05	3.96	1.40	0.50
	Female	340.11	345.5	53.88	−0.60	5.13	8.94	0.01
	Male	174.39	174.5	42.36	0.85	4.46	7.50	0.02
	<18 (Pediatric)	24.36	21.5	12.74	0.44	2.34	1.82	0.40
	18–64 (Working Age)	418.11	425	74.95	−0.07	4.10	1.85	0.40
	65+ (Geriatric)	72.03	68	15.75	0.76	3.38	3.67	0.16
**Economics** **Stressors**	Real GDP (Index)	5.25	5.22	0.14	0.17	1.78	2.40	0.30
	Unemployment Rate (%)	2.34	2.33	0.14	0.35	2.06	2.06	0.36
	CPI (Index)	6.41	6.14	0.71	0.77	2.26	4.38	0.11

Std. Dev.: standard deviation; GDP: gross domestic product; CPI: consumer price index.

**Table 3:** Overdispersion diagnostics and model selection criteria across demographic strata.

Cohort	Pearson $\left(\phi \right)$	CT Stat	CT $\left(p\right)$	Dean Stat	Dean $\left(p\right)$ (1s)	LR Stat	LR $\left(p\right)$	AIC Poisson	BIC Poisson	AIC NB	BIC NB
Overall	6.09	2.88	0.01	16.10	0.00	79.51	0.00	477.93	490.59	400.42	414.67
Female	4.01	2.96	0.01	8.72	0.00	35.53	0.00	405.39	418.06	371.86	386.11
Male	4.10	1.94	0.06	10.48	0.00	37.06	0.00	377.44	390.11	342.38	356.63
<18 (Pediatric)	1.74	0.97	0.34	1.00	0.16			241.17	253.84		
18–64 (Working Age)	5.60	2.98	0.01	14.21	0.00	68.38	0.00	456.72	469.38	390.34	404.59
65+ (Geriatric)	1.75	1.81	0.08	1.82	0.03	2.41	0.06	284.68	297.34	284.27	298.52

Pearson (ϕ): Pearson dispersion test statistic; CT stat: Cameron–Trivedi test statistic; CT *p*: probability value for Cameron–Trivedi statistic; Dean stat: Dean’s test statistic; Dean *p*(1s): one-sided probability value for Dean’s test statistic; LR stat: likelihood-ratio test statistic; LR *p*: probability value for likelihood-ratio test statistic; AIC Poisson: Akaike Information Criterion for the Poisson model; BIC Poisson: Bayesian Information Criterion for the Poisson model; AIC NB: Akaike Information Criterion for the negative binomial model; BIC NB: Bayesian Information Criterion for the negative binomial model.

**Table 4:** Parameter estimates for macroeconomic determinants of primary headache visits across demographic cohorts.

Cohort	Model	GDP	UN	CPI	COVID	Q2	Q3	Q4
Overall	NB	2.68 ***	−0.4 **	−0.35 ***	−0.12	−0.09 **	−0.03	0.01
Female	NB	2.76 ***	−0.48 **	−0.39 ***	−0.16 *	−0.06	−0.02	0.01
Male	NB	2.53 ***	−0.21	−0.27 ***	−0.02	−0.15 **	−0.05	0.01
<18 (Pediatric)	POISSON	6.08 ***	0.1	−0.39 **	−0.16	−0.24 **	0.01	−0.08
18–64 (Working Age)	NB	2.51 ***	−0.61 ***	−0.34 ***	−0.13	−0.06	−0.02	0.02
65+ (Geriatric)	POISSON	2.93 ***	0.69 ***	−0.46 ***	−0.002	−0.21 ***	−0.12	−0.03

***, ** and * represent *p* < 0.01, *p* < 0.05 and *p* < 0.10, respectively. GDP: gross domestic product; UN: unemployment; CPI: consumer price index; COVID: COVID-19-period indicator; Q2–Q4: quarter-of-year indicators with Q1 as the reference category; NB: negative binomial; HAC: heteroskedasticity- and autocorrelation-consistent. Inference is based on HAC-robust standard errors.

## Data Availability

The original contributions presented in this study are included in the article. Further inquiries can be directed to the corresponding author.

## Supplementary Materials

The following supporting information can be downloaded at: https://www.mdpi.com/article/10.3390/jcm15114181/s1, Table S1. Comparison of information criteria (AIC and BIC) for contemporaneous and lagged model specifications; Table S2. Sensitivity analysis: negative binomial estimates for cohorts retaining Poisson specification; Table S3. Residual diagnostics and statistical adequacy tests based on randomized quantile residuals (RQR); Table S4. STROBE checklist.

## Abbreviations

The following abbreviations are used in this manuscript:

GDP	Gross domestic product
CPI	Consumer price index
ICD-10	International Statistical Classification of Diseases and Health-Related Problems
ICHD-3	International Classification of Headache Disorders
OECD	Organization for Economic Cooperation and Development
TurkStat	Turkish Statistical Institute
GLM	Generalized linear models
NB	Negative binomial
KPSS	Kwiatkowski–Phillips–Schmidt–Shin
LR	Likelihood Ratio
