# Supplementary material for: The Impact of Economic Distress on Primary Headache Visits Under the Strain of the COVID-19 Pandemic: A Retrospective Study
Source: J Clin Med. 2026 May 28;15(11):4181. doi: 10.3390/jcm15114181 (PMC13258698; doi:10.3390/jcm15114181)
Supplement: Supplementary file 1 [file jcm-15-04181-s001.zip › jcm-4324043-supplementary.pdf]

**SUPPLEMENTARY NOTES**

**Table S1.** Comparison of Information Criteria (AIC and BIC) for Contemporaneous and Lagged Model Specifications

| Cohort | Model | n | AIC POISSON | BIC POISSON | AIC NB | BIC NB |
|---|---|---|---|---|---|---|
| **Overall** | **No-lags** | 36 | 477.93 | 490.59 | 400.42 | 414.67 |
| **Overall** | **1-lag** | 35 | 582.94 | 595.38 | 412.03 | 426.03 |
| **Female** | **No-lags** | 36 | 405.39 | 418.06 | 371.86 | 386.11 |
| **Female** | **1-lag** | 35 | 481.14 | 493.58 | 385.49 | 399.48 |
| **Male** | **No-lags** | 36 | 377.44 | 390.11 | 342.38 | 356.63 |
| **Male** | **1-lag** | 35 | 397.72 | 410.17 | 344.21 | 358.21 |
| **<18 (Pediatric)** | **No-lags** | 36 | 241.17 | 253.84 | | |
| **<18 (Pediatric)** | **1-lag** | 35 | 241.29 | 253.73 | 240.53 | 254.53 |
| **18–64 (Working Age)** | **No-lags** | 36 | 456.72 | 469.38 | 390.34 | 404.59 |
| **18–64 (Working Age)** | **1-lag** | 35 | 545.55 | 557.99 | 400.89 | 414.89 |
| **65+ (Geriatric)** | **No-lags** | 36 | 284.68 | 297.34 | 284.27 | 298.52 |
| **65+ (Geriatric)** | **1-lag** | 35 | 298.33 | 310.77 | 290.24 | 304.24 |

**Table S2. Sensitivity Analysis: Negative Binomial Estimates for Cohorts Retaining Poisson Specification**

| Dependent | Selected Model | GDP | UN | CPI | COVID | Q2 | Q3 | Q4 |
|---|---|---|---|---|---|---|---|---|
| **<18 (Pediatric)** | NB | 5.92*** | 0.14 | -0.36* | -0.15 | -0.23** | 0.00 | -0.09 |
| **65+ (Geriatric)** | NB | 2.96*** | 0.66*** | -0.47*** | -0.01 | -0.22*** | -0.12* | -0.03 |

**Table S3. Residual Diagnostics and Statistical Adequacy Tests Based on Randomized Quantile Residuals (RQR)**

| Dependent | Selected Model | RQR mean | RQR sd | KS stat | KS p | LB lag4 p | LB lag8 p | ACF maxlag |
|---|---|---|---|---|---|---|---|---|
| **Overall** | NB | 0.00 | 1.01 | 0.08 | 0.96 | 0.42 | 0.50 | 12 |
| **Female** | NB | 0.00 | 1.02 | 0.10 | 0.81 | 0.42 | 0.19 | 12 |
| **Male** | NB | 0.00 | 1.00 | 0.12 | 0.65 | 0.17 | 0.49 | 12 |
| **<18 (Pediatric)** | POISSON | -0.01 | 1.18 | 0.12 | 0.61 | 0.10 | 0.18 | 12 |
| **18–64 (Working Age)** | NB | 0.00 | 1.02 | 0.07 | 0.99 | 0.31 | 0.45 | 12 |
| **65+ (Geriatric)** | POISSON | 0.00 | 1.19 | 0.16 | 0.31 | 0.26 | 0.07 | 12 |

**Table S4. STROBE Checklist**

| Item | STROBE checklist domain | How addressed in the revised manuscript |
|---|---|---|
| 1 | **Title and abstract** | Title identifies the study as retrospective; Abstract summarizes background, design, data source, statistical methods, principal results and conclusion. |
| 2 | **Background/rationale** | Introduction describes economic distress, financial toxicity, primary headache burden and the private-sector utilization gap. |
| 3 | **Objectives** | Introduction states the research question and hypothesis in the final paragraph. |
| 4 | **Study design** | Materials and Methods identifies a single-center, retrospective, ecological quarterly time-series design. |
| 5 | **Setting** | Data Collection reports the private tertiary hospital setting, Turkey's Black Sea region and the 2016 Q1-2024 Q4 study period. |
| 6 | **Participants** | Data Collection describes eligible hospital-visit records, exclusions, final sample size and demographic stratification; matched-study elements are not applicable. |
| 7 | **Variables** | Structural Framework and Macroeconomic Data Sources define visit counts, GDP, CPI, unemployment and the COVID-period indicator. |
| 8 | **Data sources/measurement** | Data Collection describes hospital information system records, ICD-10 coding, physician notes, ICHD-3 framework and TurkStat macroeconomic data. |
| 9 | **Bias** | Methods and Limitations address retrospective single-center design, ecological aggregation and unavailable patient-level socioeconomic information. |
| 10 | **Study size** | Data Collection reports 18,589 raw records, 67 exclusions and 18,522 eligible records; quarterly aggregation produced 36 observations. |
| 11 | **Quantitative variables** | Covariate specification explains logarithmic macroeconomic variables, raw count outcomes and quarterly timing. |
| 12 | **Statistical methods** | Modelling framework describes Poisson/NB GLMs, overdispersion diagnostics, model selection, HAC inference, seasonality controls and residual diagnostics. |
| 13 | **Participants/results flow** | Results and Figure 1 report exclusions, final analytic dataset and demographic strata. |
| 14 | **Descriptive data** | Results Table 1 and Table 2 provide demographic distribution and descriptive statistics. |
| 15 | **Outcome data** | Results summarize quarterly primary headache visit counts overall and by subgroup. |
| 16 | **Main results** | Table 4 and accompanying text report GDP, unemployment, CPI, COVID-period and seasonal coefficients across cohorts. |
| 17 | **Other analyses** | Supplementary Tables S1-S3 report lag sensitivity, NB sensitivity for Poisson-selected cohorts and RQR diagnostics. |
| 18 | **Key results** | Discussion opens by distinguishing private-sector utilization from underlying headache burden and summarizes the main interpretation. |
| 19 | **Limitations** | Discussion/Limitations describes single-center private-hospital data, ecological design, unobserved patient-level variables and lack of public-sector data. |
| 20 | **Interpretation** | Discussion interprets results in relation to financial toxicity, prior economic-stress literature and primary headache psychosomatic frameworks. |
| 21 | **Generalisability** | Limitations and Policy Implications state that generalizability to public hospitals and other regions is limited. |
| 22 | **Funding** | Back matter states that the research received no external funding. |
